# Eco-friendly cucurbituril-based potentiometric sensors for selective quantification of ipratropium bromide in pharmaceuticals and human plasma

**DOI:** 10.1007/s00216-025-06265-5

**Published:** 2025-12-22

**Authors:** Samia A. Tawfik, Amr M. Mahmoud, Aya T. Soudi

**Affiliations:** Pharmaceutical Analytical Chemistry Department, Faculty of Pharmacy, Cairo University, Kasr El-Aini Street, Cairo, 11562 Egypt

**Keywords:** Ipratropium bromide, Green analytical chemistry, Graphene, Glassy carbon electrode, Potentiometric sensors, Cucurbituril

## Abstract

This work presents a novel and innovative approach for the quantification of ipratropium bromide (IPBr), a quaternary ammonium drug widely used in the management of asthma and chronic obstructive pulmonary disease (COPD). IPBr is administered via inhalation. Despite its localized action in the lung and reduced systemic side effects, accurate quantification of IPBr in pharmaceutical formulations and biological matrices remains critical, especially in scenarios of accidental ingestion. The proposed method utilizes cucurbituril as a selective molecular receptor-type ionophore in potentiometric-based ion-selective electrodes (ISEs), offering a green, portable, and cost-effective alternative to traditional analytical techniques. Cucurbituril’s unique hydrophobic cavity and polar portals enable highly selective encapsulation of IPBr through host–guest chemistry, enhancing sensitivity and ensuring accurate detection in complex matrices such as pharmaceutical preparations and human plasma. Two sensor platforms were developed: a liquid-contact and a solid-contact electrode. The solid-contact electrode was fabricated by drop-casting the membrane components onto a graphene-modified glassy carbon electrode. The solid-contact design eliminates the limitations of liquid-contact systems, while improving mechanical stability and compatibility with miniaturized devices. Both sensors demonstrated excellent Nernstian responses (around 57 mV/decade), rapid response times of 5 s, wide linear ranges (1 × 10^–6^–1 × 10^–2^ M), and high selectivity against interfering substances. The method was successfully validated for IPBr quantification in Atrovent® inhalers and spiked human plasma, demonstrating recoveries of 98.10–100.08% without any interference from excipients or biological matrices. This study presents cucurbituril-based solid-contact sensors as reliable and environmentally friendly tools for therapeutic drug monitoring and pharmaceutical analysis. The method’s sustainability was evaluated using the Analytical Eco-Scale, AGREE, and RGB models, confirming its sustainable performance. The developed sensors also contribute to several United Nations Sustainable Development Goals (UN–SDGs), including those related to health, clean water, sustainable production, and innovation.

**Graphical Abstract:**

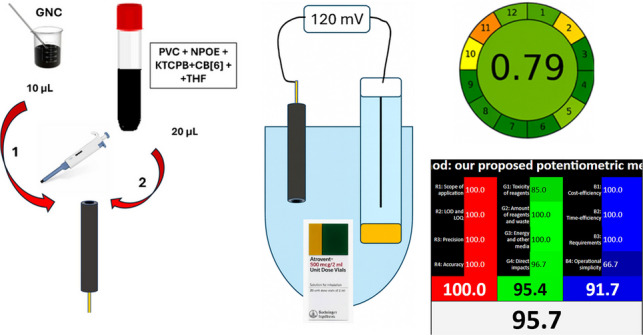

**Supplementary Information:**

The online version contains supplementary material available at 10.1007/s00216-025-06265-5.

## Introduction

Asthma is the most prevalent chronic respiratory condition in the world. It is known by inflammation and narrowing of the small airways accompanied by wheezing, shortness of breath, chest tightness, and coughing. Asthma pathophysiology consists of genetic, environmental, and immunologic components. The triggering factors are allergens, air pollution, respiratory infections, and exercise can exacerbate asthma symptoms by causing airway inflammation, increased mucus production followed by bronchoconstriction and finally airway obstruction [1]. Management of asthma typically involves a combination of pharmacological and non-pharmacological strategies. Non-pharmacological strategies involve trigger avoidance, breathing exercises and patient education. The first line in pharmacological strategies is the use of bronchodilators, such as short-acting beta-agonists (SABAs), offer rapid relief by relaxing airway muscles, while a combination of long-acting muscarinic antagonist (LAMA) and inhaled corticosteroids (ICS) are the cornerstone of long-term control, for managing bronchospasms associated with chronic obstructive pulmonary disease (COPD) [1].

**Fig. 1:**
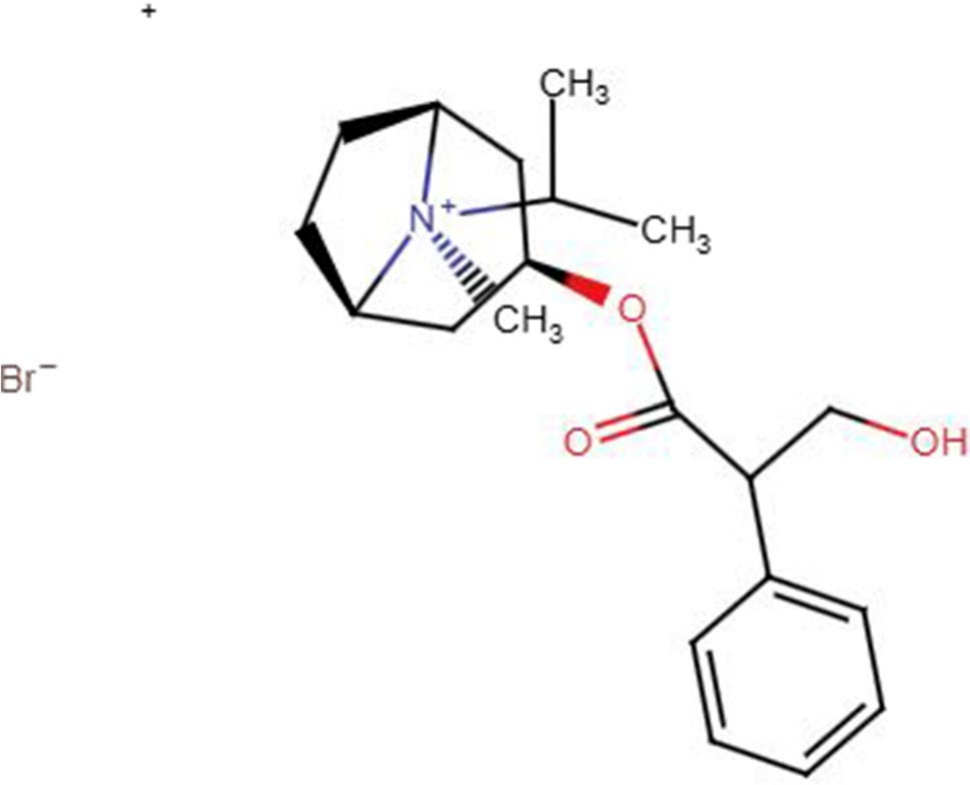
Chemical structure of ipratropium bromide (IPBr)

Ipratropium bromide (IPBr) (Fig. 1) is a long-acting muscarinic antagonist (LAMA). It is administered generally by inhalation with local action without any significant systemic side effects. Inhaled IPBr is an add-on therapy to inhaled corticosteroids for the management of severe asthma exacerbations [2]. Chemically, it is (1R,3R,5S)−3-[(3-hydroxy-2-phenylpropanoyl)oxy]−8-methyl-8-(propan-2-yl)−8-azabicyclo[3.2.1]octan-8-ium bromide. The log P of IPBr is approximately −1.8 [2], indicating that it is highly hydrophilic and has greater solubility in water compared to lipids, which greatly minimizes systemic absorption when administered via inhalation, reducing its side effects and making it effective for local action in the lungs. Despite being intended for local action, most of the administered IPBr dose is swallowed and passes through the gastrointestinal tract rather than depositing in the bronchial mucosa [3]. The swallowed portion results in systemic absorption through the GI route. Consequently, plasma IPBr level reflects its oral absorption highlighting the importance of developing an appropriate analytical technique in monitoring and quantifying IPBr.

Various methods for the quantification of IPBr have been reported in the literature including high-performance liquid chromatography (HPLC) [4–8], spectrophotometry [9–12], and potentiometry [13–15]. While these techniques are well-established, the incorporation of cucurbituril as host molecule forms a highly stable host-guest complex with IPBr, enhancing sensitivity and selectivity in electrochemical detection [16].

Cucurbiturils (CBs) have been effectively used in electrochemical sensors to enhance sensitivity and selectivity, particularly for detecting quaternary ammonium compounds like IPBr, due to their hydrophobic cavity and polar portals [16]. This selective encapsulation and unique host–guest chemistry enable the preconcentration of the target analyte near the electrode surface, improving the sensor’s response and ensuring accurate detection and quantification of drugs [16].

Liquid-contact sensors, while effective, present challenges including dependence on liquid electrolytes, limited portability, and susceptibility to environmental factors. So, transitioning from liquid-contact to solid-contact sensors using cucurbiturils represents an advancement in pharmaceutical sensing. This shift not only eliminates the need for liquid phases but also enhances sensor stability, portability, and usability. Solid-contact sensors leveraging cucurbiturils offer a promising pathway to robust, miniaturized, and environmentally sustainable solutions for pharmaceutical analysis of quaternary ammonium drugs like IPBr [17–19].

Solid-based sensing platforms have shown significant promise in clinical applications, especially cucurbituril-based solid sensors which shows a great promise for clinical applications, particularly in plasma drug analysis and therapeutic drug monitoring (TDM) for quaternary ammonium containing drugs. These areas require high specificity and sensitivity to accurately monitor drug concentrations in patient plasma samples, ensuring that therapeutic levels are maintained to improve patients’ outcomes and to optimize treatment regimens [19, 20]. To further improve transduction performance, we employed graphene as the solid-contact ion-to-electron transducer. Graphene brings advantages like its high electrical conductivity and large surface area which yield high double-layer capacitance and remarkably stable potentiometric signals, with minimal drift [21]. Moreover, its strong hydrophobicity prevents disruptive water layer formation at the interface, enhancing the sensor’s robustness and long-term stability [21]. By integrating cucurbituril host–guest specificity with graphene’s superior transduction properties, our platform delivers fast, reliable, and precise monitoring of IPBr in complex biological matrices.

In recent years, the pharmaceutical industry and regulatory bodies have increasingly emphasized health, environmental, and safety considerations in the development and application of analytical methods. This approach is consistent with the core concepts of Green Analytical Chemistry (GAC), a global trend aimed at creating eco-friendly analytical approaches. GAC promotes eco-friendly approaches by minimizing the use of toxic solvents, reducing waste, and ensuring sustainable practices [22–28]. Tools like the Analytical Eco-Scale [29] and AGREE algorithm [30] are used to assess the greenness of the proposed methods. However, achieving greenness should not come at the expense of analytical functionality. Therefore, the concept of White Analytical Chemistry (WAC) has emerged as an extension of GAC. WAC integrates environmental, analytical, and practical aspects, ensuring a balance between sustainability and performance [31, 32]. A key feature of both GAC and WAC is the miniaturization of analytical devices, which offers faster analysis, reduced reagent use, cost savings, and portability for on-site applications [33, 34]. Together, GAC and WAC provide a comprehensive framework for developing efficient, eco-friendly, and functional analytical methods in pharmaceutical research and quality control processes.

In this work, we introduce a novel approach for the quantification of IPBr, using cucurbituril as selective molecular receptor-type ionophore via green, portable, trustworthy and budget-friendly potentiometric based ion-selective electrodes. The method employs both liquid-contact and solid-contact platforms and is applicable to bulk drug, pharmaceutical formulation, and spiked human plasma samples.

## Experimental

### Instruments

Potentiometric measurements were conducted using a double-junction Ag/AgCl reference electrode sourced from Merck KGaA, Darmstadt, Germany. A Jenway 3510 pH meter (Staffordshire, UK) was employed for pH adjustments and recording data. Continuous stirring during measurements was maintained using a Stuart US152 magnetic stirrer, also manufactured in Staffordshire, UK.

### Chemicals, reagents, and pharmaceutical formulation

Ipratropium bromide was kindly supplied by Boehringer Ingelheim Pharma GmbH & Co, Egypt. Its purity was certified to be 100.17% ± 0.69. Atrovent® Inhaler CFC-free 500 µg/2 mL inhalation solution is obtained from the local Egyptian market. High molecular weight polyvinyl chloride (PVC), tetrahydrofuran (THF), xylene, potassium tetrakis(4-chlorophenyl) borate (KTCPB), Cucurbit[6]uril (CB[6]), nitrophenyl octyl ether (NPOE), and Ipratropium impurity C (3-hydroxy-2-phenyl propanoic acid) were supplied from Sigma-Aldrich (Steinheim, Germany). Graphene nanoplatelets, with a thickness of 6–8 nm and an average diameter of 5 µm, were purchased from Strem Chemicals INC. (Newburyport, USA). Inorganic reagents including potassium chloride, sodium chloride, sucrose, urea, lactic acid, dipotassium hydrogen phosphate, and potassium dihydrogen phosphate were sourced from Merck (Darmstadt, Germany). Deionized water was provided by SEDICO Pharmaceuticals Co. (6th October City, Egypt), while human plasma samples were obtained from the Holding Company for Biological Products and Vaccines (VACSERA), Egypt.

### Standard solutions

A 1.0 × 10^–2^ M IPBr stock solution was prepared in a 50 mL volumetric flask using deionized water. Working standard solutions (1.0 × 10^–2^ to 1.0 × 10^–7^ M) were obtained by diluting the stock with phosphate buffer (pH 6.0). Interference test solutions, containing IPBr impurity C, KCl, NaCl, sucrose, urea, and lactic acid, were also prepared in the same buffer from 1.0 × 10^–2^ M to 1.0 × 10^–5^ M. All solutions were stored at 4 °C and remained stable for at least four weeks.

### Method development, optimization, and validation procedures

#### Preparation of graphene nanocomposite (GNC)

A graphene-based nanocomposite was prepared via a solution dispersion method adapted from Li et al. [35]. First, 10 mg of graphene nanoplatelets was ultrasonically suspended in 1 mL xylene for 5 min to achieve a uniform suspension. In parallel, 95 mg of PVC was dissolved in 3 mL THF, and 0.20 mL NPOE was added as a plasticizer. These two solutions were then combined and subjected to a further 10 min of ultrasonication to ensure uniform integration of graphene within the PVC matrix. This procedure provided reproducible, stable nanocomposites suitable for sensor fabrication.

#### Fabrication and assembly of the liquid membrane sensor

The ion-selective membrane (ISM) was prepared in a 5-cm-diameter Petri dish by combining 190 mg of PVC with 10 mg KTCPB and 20 mg CB[6] followed by the addition of 0.4 mL NPOE to form a uniform slurry. Six milliliters THF was added to this slurry in the Petri dish and stirred thoroughly until completely homogeneous. A filter paper was placed over the Petri dish, and the setup was left to dry at room temperature overnight. The resulting membrane had an approximate thickness of 0.1 mm. From this membrane, a 5-mm-diameter circular disc was carefully cut and fixed to an elastic PVC tip using THF. The inner compartment of the electrode was filled with equal volumes of 1.0 × 10–2 M KCl and 1.0 × 10–2 M IPBr solutions (total volume ≈ 0.5 mL). A 1 mm Ag/AgCl wire was inserted into this filling solution to serve as the internal reference. Prior to use, the sensor underwent conditioning by soaking in a 1.0 × 10^–3^ M IPBr solution for 24 h.

#### Fabrication of the solid-contact sensor

Ten microliters of a graphene nanocomposite dispersion was drop-cast onto the glassy carbon electrode. Once dry, 20 µL of the ISM layer (mixture of 400 μL of NPOE, 190 mg of PVC, 20 mg CB[6], and 10 mg KTCPB, and all dispersed in 6 mL of THF) was subsequently drop-cast on top. The prepared sensor was conditioned for 24 h by soaking in 1 × 10^–4^ M IPBr solution before conducting any measurements.

#### Potentiometric measurements and sensors calibration

Potentiometric measurements for both liquid- and solid-contact electrodes were carried out using a Jenway 3510 digital ion analyzer paired with a double-junction Ag/AgCl external reference electrode. For each measurement, the prepared sensor was immersed simultaneously with the external reference electrode in the solutions. Quantitative assays were conducted using IPBr standard solutions prepared in pH 6.0 phosphate buffer. Calibration curves were generated by plotting the measured potential against the logarithmic concentration of IPBr over the range of 1 × 10^–7^ to 1 × 10^–2^ M. This range was utilized to construct the calibration curve and determine the limit of detection (LOD). The electrode demonstrated a linear response over the concentration range of 1 × 10^–6^ to 1 × 10^–2^ M which was used as the method calibration range. The performance of the electrodes was evaluated in accordance with the guidelines set by the International Union of Pure and Applied Chemistry (IUPAC) [36].

#### Effect of pH and interfering ions on sensor’s performance

The effect of pH variation on the potential readings was evaluated by immersing the developed sensors in a 1 × 10^–3^ M and 1 × 10^–4^ M IPBr solution across a pH range of 2.0 to 12.0. The potential corresponding to each pH value was recorded.

The potentiometric selectivity coefficient of the sensor was determined using the separate solutions method [36]. The selectivity was tested in the presence of IPBr impurity C and various potential interfering substances which are prevalent in biological fluids. It was calculated by substituting the potential response to IPBr and that produced by the same concentration of different interfering species into the Nicolsky-Eisenman equation [36]. Furthermore, the potential of both IPBr and some interfering ions was assessed across a concentration range from 1.0 × 10^–5^ to 1.0 × 10^–2^ M, following IUPAC guidelines [36].

#### Water layer test

The stability of the graphene-modified glassy carbon solid-contact sensor was evaluated against a bare glassy carbon electrode by monitoring potential shifts after immersing the electrode in 1.00 × 10^–4^ M IPBr for 1 h, then in 1.00 × 10^–3^ M trazodone for 1 h, and finally returned to the 1.00 × 10^–4^ M IPBr solution for an additional hour. This protocol was specifically designed to assess the potential formation of a water layer at the electrode interface.

#### Potentiometric quantification of IPBr in Atrovent® inhaler and in human plasma

To prepare a 3 × 10^–4^ M IPBr solution, an accurately measured volume of the inhaler formulation was transferred into a 10-mL volumetric flask and brought to the mark with phosphate buffer (pH 6.0). Potentiometric measurements were conducted as outlined earlier, under the “Potentiometric measurements and sensors calibration” section. Drug concentrations were determined using the appropriate regression equation derived from the calibration curve.

For plasma, to prepare a range of IPBr concentrations in plasma (1.0 × 10^–6^ M to 1.0 × 10^−2^ M), various aliquots of IPBr working standard solutions were mixed with 1 mL of IPBr-free human plasma in separate 10-mL volumetric flasks. The flasks were then filled to the mark with phosphate buffer at pH 6.0. Following the same procedure, quality control (QC) samples were prepared at three concentration levels: low QC (LQC) at 5.0 × 10^−6^ M, medium QC (MQC) at 5.0 × 10^–5^ M, and high QC (HQC) at 5.0 × 10^–4^ M. Measurements were conducted as outlined in the “Potentiometric measurements and sensors calibration” section. The recorded potential values were used to generate a calibration curve for the sensor, and sample concentrations were calculated using the derived regression equation.

## Results and discussion

The design of potentiometric sensors that are sensitive, selective, and economically efficient can significantly enhance drug screening processes in various settings. This study focuses on using cucurbituril as an ionophore to construct two sensors that are not only easy to prepare but also highly selective to determine IPBr in various matrices. To achieve this, a two-step optimization approach was employed. Initially, a liquid-contact electrode was implemented, followed by drop-casting the membrane components onto a graphene-modified glassy carbon electrode. Through comparative stability testing against a bare glassy carbon electrode, the graphene-modified sensor demonstrated superior potential stability. So, a graphene-based solid-contact sensor was selected for all further measurements, where it effectively eliminates the limitations of traditional liquid-contact systems.

### Rationale for plasticizer selection

Among dibutyl sebacate (DBS), dioctyl phthalate (DOP), and nitrophenyl octyl ether (NPOE), NPOE exhibits a higher dielectric constant compared to DBS and DOP. This characteristic property improves the solubility and mobility of quaternary ammonium ions like IPBr within the membrane, improving both ionic exchange and sensor performance. NPOE polarity aligns well with the hydrophilic nature of quaternary ammonium compounds, promoting better interaction and ion transport within the membrane. Moreover, NPOE offers effective plasticization, which contributes to membrane homogeneity, mechanical stability, and improved electrochemical responses [37, 38]. In contrast, DBS and DOP, being less polar, are less effective at supporting the ionic interactions of IPBr, making them less suitable for sensors targeting quaternary ammonium compounds. Thus, NPOE is the most suitable plasticizer compared to the other options and is therefore the chosen plasticizer for the IPBr sensor.

### Rationale for selecting CB[6] ionophore in sensors fabrication

The performance of ion-selective electrodes (ISEs) depends on various conditions, including the incorporated ionophore in the fabricated membrane. Receptor-type ionophores create selective recognition sites for specific ions, forming reversible complexes with the target ion at the membrane interface, which enhances the selectivity and sensitivity of the sensor [39]. IPBr is a quaternary ammonium compound, so the best ionophore for its potentiometric determination is the one that selectively interacts with the quaternary ammonium group in IPBr, enhancing selectivity and sensitivity. Calix[4]arene, β-CD, and CB[6] were separately used to choose the most compatible one with IPBr. CB[6] has shown superior selectivity and sensitivity for IPBr, especially when used in conjunction with a plasticizer like nitrophenyl octyl ether (NPOE). Their unique cavity structure ensures effective interaction with the quaternary ammonium moiety of IPBr.

### Sensors fabrication

Since IPBr exists as a cation over a wide pH range, we incorporated KTCPB, an ion-exchanger with a cation exchange capacity, to substitute its original counter ion with IPBr. The sensor was conditioned for 24 h in 1 × 10^–4^ M IPBr solution. The resulting Nernstian slope, which is about 60 mV/decade, confirms that IPBr functions as a monoionic species. The liquid-contact electrode is associated with some limitations, such as the evaporation of the inner filling solution, ion flux through the membrane, and the inability to miniaturize the design effectively. These challenges decrease long-term stability, hinder portability, and limit its application in modern analytical techniques. To overcome these limitations, we develop the solid-contact electrode. Its design eliminates the need for an inner filling solution, improves mechanical stability, and enhances compatibility with miniaturized and portable devices. By adopting this approach, we successfully advanced the development of a robust sensor for the precise and selective determination of IPBr in different samples, meeting the requirements of clinical and therapeutic drug monitoring applications.

### Sensors performance characteristics

**Fig. 2:**
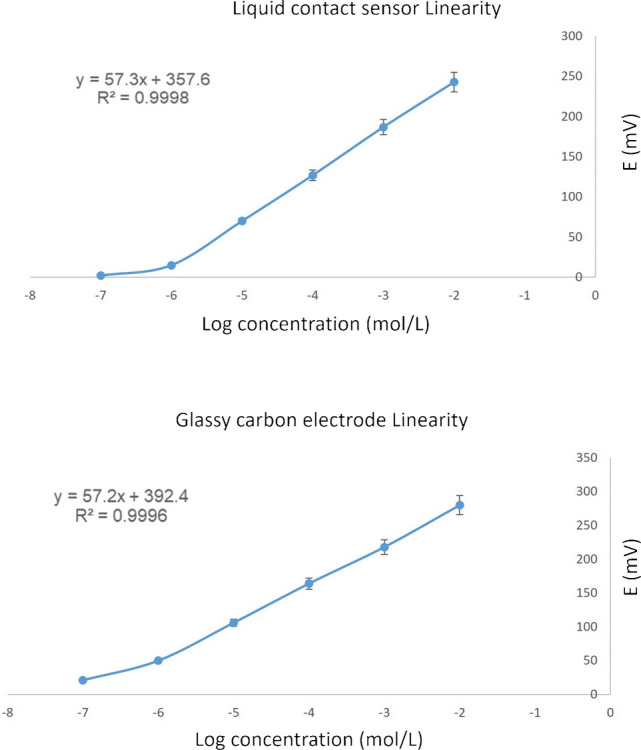
Profile of the potential in *mV* versus log concentrations in *mol/L* of IPBr for both liquid-contact sensor and graphene-modified glassy carbon electrode

The electrochemical behavior of the proposed sensors for IPBr was assessed following IUPAC recommendations [36]. As shown in Fig. 2, both sensors exhibited a linear response within the 1 × 10^–6^ to 1 × 10^–2^ M concentration range, with slopes of 57.3 mV/decade for the liquid-contact configuration and 57.2 mV/decade for the graphene-based solid-contact sensor. Linear regression equations were established from the respective calibration curves. Key performance metrics—such as linearity, precision, accuracy, stability, and robustness—are outlined in Table 1. During validation, both sensors showed stable readings with fluctuations within ± 1.0 mV. The response time, defined by the duration needed to stabilize after a tenfold IPBr concentration shift, was approximately 5 s. The stability of the fabricated sensors was evaluated over several weeks under ambient conditions. Sensors were kept dry when not in use. Repeated measurements during this period showed negligible changes in potential response and reproducibility, confirming that the solutions and sensors maintained consistent performance suitable for routine use. The liquid-contact sensor remained stable for 3 weeks, while the graphene-modified solid-contact variant retained functionality for up to 8 weeks. These results confirm the sensors’ reliability and suitability for IPBr analysis.

**Table 1:** Electrochemical response characteristics of the investigated sensors

**Parameters**		**Liquid-contact sensor**	**Solid-contact sensor**
**Linearity**	Range (M)	1.0 × 10^–6^–1.0 × 10^–2^	1.0 × 10^−6^–1.0 × 10^−2^
	Slope	57.3	57.2
	Intercept	357.6	392.4
	Correlation coefficient (r)	0.9998	0.9996
**LOD**^**a**^** (M)**		7.0 × 10^−7^	6.0 × 10^–7^
**Accuracy**^**b**^ (mean ± SD)		99.60 ± 1.8	99.33 ± 1.8
**Precision (RSD %)**			
Repeatability^c^ Intermediate precision^d^		1.152 1.7	0.766 1.1
**Robustness**^**e**^** (RSD %)**		0.963	1.059
**Stability (days)**		21	60
**Response time (sec)**		20	5

^a^LOD (limit of detection) was measured by interception of the extrapolated arms of the calibration graph

^b^Average of three determinations

^c^The intraday precision, average of three concentrations repeated three times within the day

^d^The interday precision, average of three concentrations repeated three times on three successive days

^e^Robustness, variations in method parameters; pH of the medium (± 0.4) and agitation rate (± 6 rpm)

### Water layer test

**Fig. 3:**
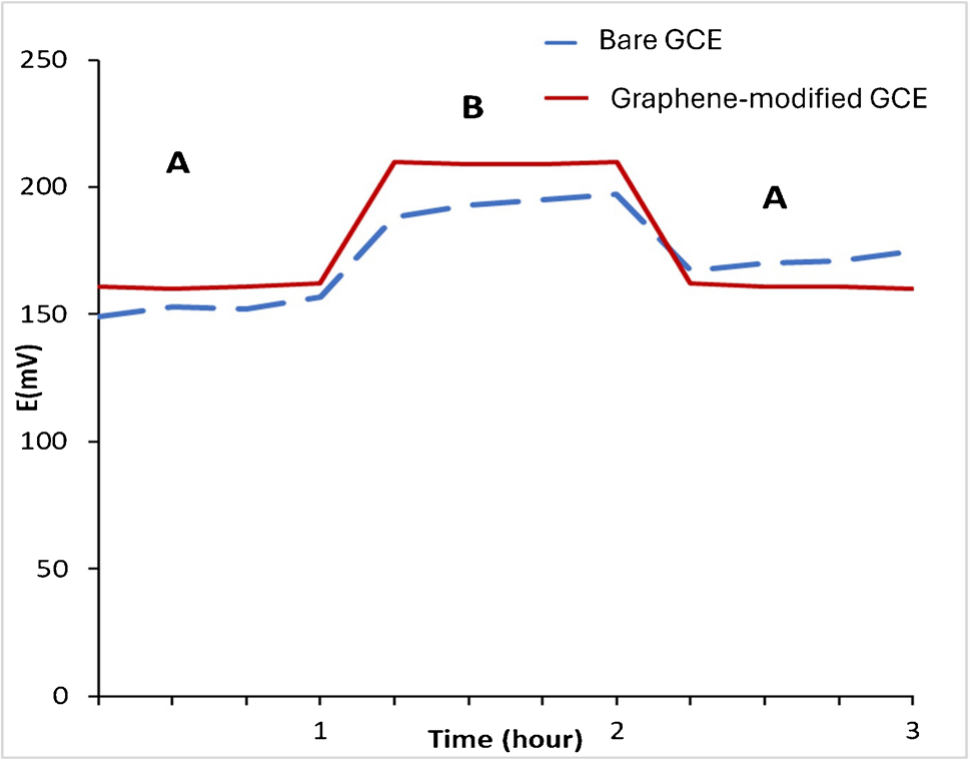
Water layer test for bare and graphene-modified glassy carbon electrode. Measurements were recorded in 1.00 × 10^–4^ M IPBr and 1 × 10^–3^ M Trazodone (B)

The stability of the graphene-modified glassy carbon electrode was compared to that of the bare glassy carbon electrode using the water layer test. In this test, each electrode was immersed sequentially in 1.00 × 10^–4^ M IPBr, then in 1.00 × 10^–3^ M trazodone, and finally returned to the IPBr solution. Trazodone was selected as a model cationic drug for this test as it represents a positively charged, moderately lipophilic species similar in behavior to IPBr. The graphene-enhanced sensor consistently maintained stable potential, demonstrating rapid, selective responses to IPBr and clear reversibility when changed with trazodone. In contrast, the bare electrode exhibited potential drift, indicating the formation of an undesirable water layer (Fig. 3). This comparison confirms that graphene’s hydrophobicity effectively prevents water layer formation and provides excellent signal stability. So, the graphene-modified solid-contact electrode was chosen as the transduction platform for all subsequent measurements, offering superior reliability, specificity, and robustness.

### pH and selectivity studies of the fabricated sensors

**Fig. 4:**
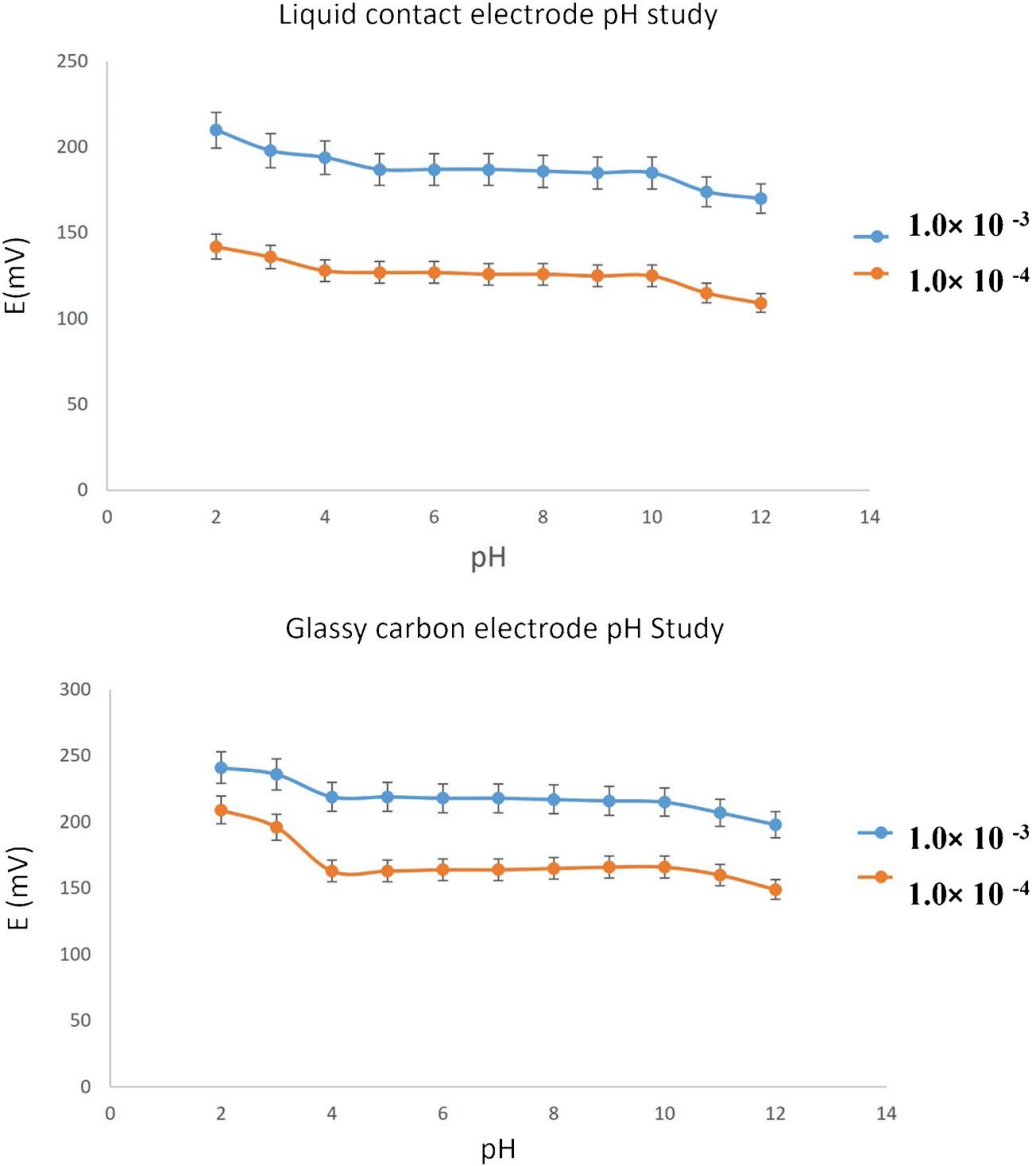
Effect of pH on the performance of both liquid-contact sensor and graphene-modified glassy carbon electrode in 10^–3^ and 10^–4^ mol/L IPBr

To adjust the pH, small volumes of 1 N hydrochloric acid or 1 N sodium hydroxide were added as required. The impact of pH on sensor performance was evaluated using IPBr concentrations of 10^–4^ M and 10^–3^ M across a pH range from 2.0 to 12.0 (Fig. 4). Both liquid- and solid-contact sensors exhibited stable potential responses within the 4.0–10.0 pH window, confirming that their performance is largely independent of pH in this range. This stability arises from the fact that IPBr, being a quaternary ammonium compound, carries a permanent positive charge and remains fully ionized across the entire pH range; therefore, it is detected as a monovalent ionic species throughout the entire pH scale.

**Fig. 5:**
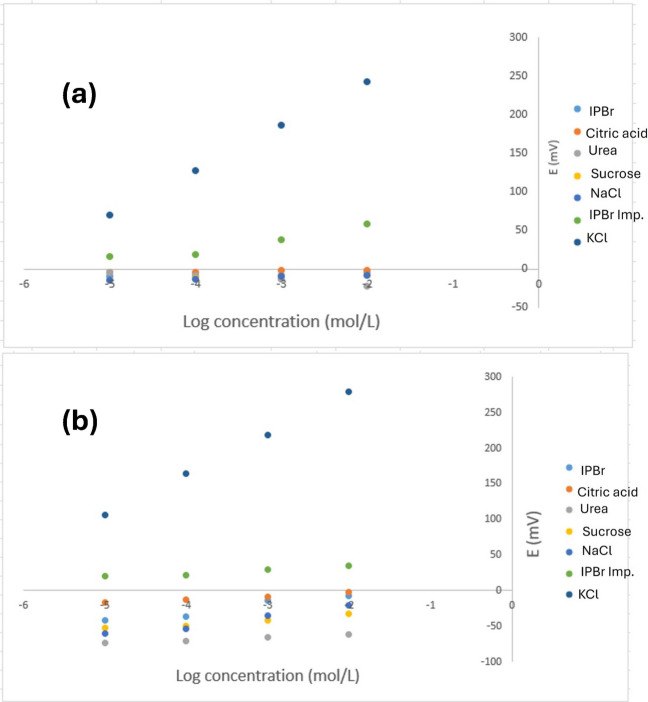
Calibration curves obtained for IPBr and some relevant concentrations of interfering ions using (**a**) liquid-contact sensor and (**b**) solid-contact sensor

The potentiometric selectivity coefficient measures the degree to which an interfering ion, commonly found in pharmaceutical formulations with IPBr (such as excipients or impurities), affects the sensor’s response to IPBr. The selectivity of the two fabricated sensors was evaluated against different interfering ions, including impurity C of IPBr, using the separate solution method (SSM) with the results summarized in Table 2 [36]. The results reveal that the solid-contact sensor exhibits higher selectivity to IPBr compared to the liquid-contact sensor, due to its stable solid-state interface, which eliminates the liquid layer typically susceptible to ion exchange and interference. Furthermore, the hydrophobic properties of the solid contact minimize fouling and electrical noise, leading to lower selectivity coefficients and enhanced reliability in complex pharmaceutical matrices [40]. Both sensors were tested with various concentrations of IPBr impurity C and other interfering ions. Calibration graphs presented in Fig. 5 display non-Nernstian slopes for the interfering ions, confirming negligible interference in IPBr determination. Additionally, the low selectivity coefficient values indicate that the sensor membranes are minimally affected by coexisting ions. These findings ensure accurate and reliable quantification of IPBr, even in the presence of potential interfering substances.

**Table 2:** Selectivity coefficients of the fabricated sensors towards the interfering ions

**Interfering ion (1.0 × 10**^**–3**^ M)*	**Selectivity coefficients (K**^**Pot**^**)**	
	**Liquid-contact sensor**	**Solid-contact sensor**
Sodium chloride	3.98 × 10^–4^	8.71 × 10^–5^
Potassium chloride	3.80 × 10^–4^	3.80 × 10^–5^
Citric acid	5.04 × 10^–4^	1.07 × 10^–4^
Urea	3.24 × 10^–4^	1.07 × 10^–5^
Sucrose	3.63 × 10^–4^	2.82 × 10^–5^
IPBr impurity C	2.51 × 10^–3^	5.01 × 10^–4^

*Average of three determinations

### Determination of IPBr in Atrovent® inhaler dosage form

The fabricated sensors were successfully used for the quantification of IPBr in Atrovent**®** inhaler. The average percentage recoveries were 98.10 ± 2.03 for the liquid-contact sensor and 100.08 ± 0.63 for the graphene-modified glassy carbon electrode. The dosage form excipients showed no interference, as discussed in sensor selectivity. So, IPBr determination was performed without the need for prior processing or extraction. The method’s validity was confirmed by statistical comparison with a previously published assay using unpaired t and F tests [4]. The calculated t- and F-values were below the critical thresholds, validating the null hypothesis, indicating no significant difference in accuracy or precision between the proposed sensors and the reference method (Table 3).

**Table 3:** Statistical comparison of the results obtained by the developed methods and reported method for IPBr determination in its pharmaceutical formulation

**Item**	**Liquid-contact sensor**	**Solid-contact sensor**	**Reported method**^**a**^
Mean	98.10	100.08	100.02
SD	2.03	0.63	0.97
Variance	4.121	0.397	0.941
n	5	5	5
Degree of freedom	4	4	4
Student’s t-test (2.571)^b^	1.908	0.116	
F value (6.388)^b^	4.380	2.337	

^a^RP-HPLC method using C18 column (250 × 4.6 mm, 5 µ) with mobile phase composed of 0.1% trifluoroacetic acid and acetonitrile (70:30 v/v) at a flow rate of 1.0 mL/min and UV detection wavelength at 210.0 nm

^b^Figures in parentheses are the corresponding tabulated values for t and F at *p* = 0.05

### Determination of IPBr in spiked human plasma

It was mentioned that ipratropium bromide is administered via inhalation, with minimal systemic absorption occurring through the small amount inadvertently ingested via the oral route. However, in cases of accidental ingestion, particularly by a child or an adult, monitoring the plasma concentration of the drug becomes critical. Such incidents can lead to elevated plasma levels, which may lead to undesirable side effects, which makes IPBr quantification necessary in plasma samples to evaluate the amount absorbed, guide appropriate medical intervention, and ensure patient safety.

The fabricated sensors were effectively used to measure IPBr concentrations in human plasma at three levels: LQC at 5.0 × 10^−6^ M, MQC at 5.0 × 10^−5^ M, and HQC at 5.0 × 10^−4^ M. In line with FDA recommendations [41], quality control samples were analyzed in triplicate within the same day and across three consecutive days to assess the accuracy and precision of the proposed sensors for IPBr determination in plasma samples (Table 4). Blank plasma samples showed no detectable potential response, confirming the absence of endogenous interference.

**Table 4:** Precision and accuracy of IPBr determination in spiked human plasma using the fabricated liquid- and solid-contact sensors

Nominal concentration (M)	Intraday (n = 9)				Interday (n = 9)			
	Liquid-contact sensor		Solid-contact sensor		Liquid-contact sensor		Solid-contact sensor	
	% Accuracy	%RSD	% Accuracy	%RSD	% Accuracy	%RSD	% Accuracy	%RSD
QCL^a^ (5.0 × 10^−6^)	100.03	0.89	102.30	0.94	100.29	1.94	99.63	2.00
QCM^b^ (5.0 × 10^−5^)	98.98	1.043	97.60	1.21	101.12	1.06	101.31	1.59
QCH^c^ (5.0 × 10^−4^)	97.72	1.321	100.21	1.834	98.17	1.33	98.17	1.71

^a^Low quality control

^b^Medium quality control

^c^High quality control

*Average of three determinations

### Green analytical perspective; evaluation of the greenness, whiteness and UN–SDGs integration of the proposed potentiometric methods

The greenness of an analytical method reflects its overall environmental impact, and electrochemical methods align with green chemistry principles. Electrochemical analysis has emerged as one of the most promising eco-friendly techniques due to its inherent advantages, such as the elimination of complex sample pre-treatment steps and the potential for real-time, in situ analysis without the need for hazardous chemicals or solvents. The miniaturized design utilizes only microliter-level solution volumes and operates without the need for high-energy instrumentation, thereby improving energy efficiency and reducing the overall analytical carbon footprint. Moreover, the electrode’s long operational lifetime and stable response reduce material waste and the need for frequent recalibration. These features comply with key GAC principles, including safe solvent and reagent usage, waste minimization, and energy efficiency [22, 42–45]. We assessed the greenness of our sensors using the Analytical Eco‑Scale and AGREE metrics, which evaluate factors like energy usage, waste generation, and chemical hazards. To take sustainability further, we applied WAC principles using the RGB 12 model, which integrates eco-friendliness (green), analytical performance (red), and operational practicality (blue). The results in Table 5 affirm the method’s outstanding environmental profile and overall whiteness, supporting its suitability for green pharmaceutical analysis. Moreover, the proposed sensors were evaluated with respect to the United Nations Sustainable Development Goals (UN–SDGs) to evaluate their alignment with global sustainability principles.

**Table 5:**
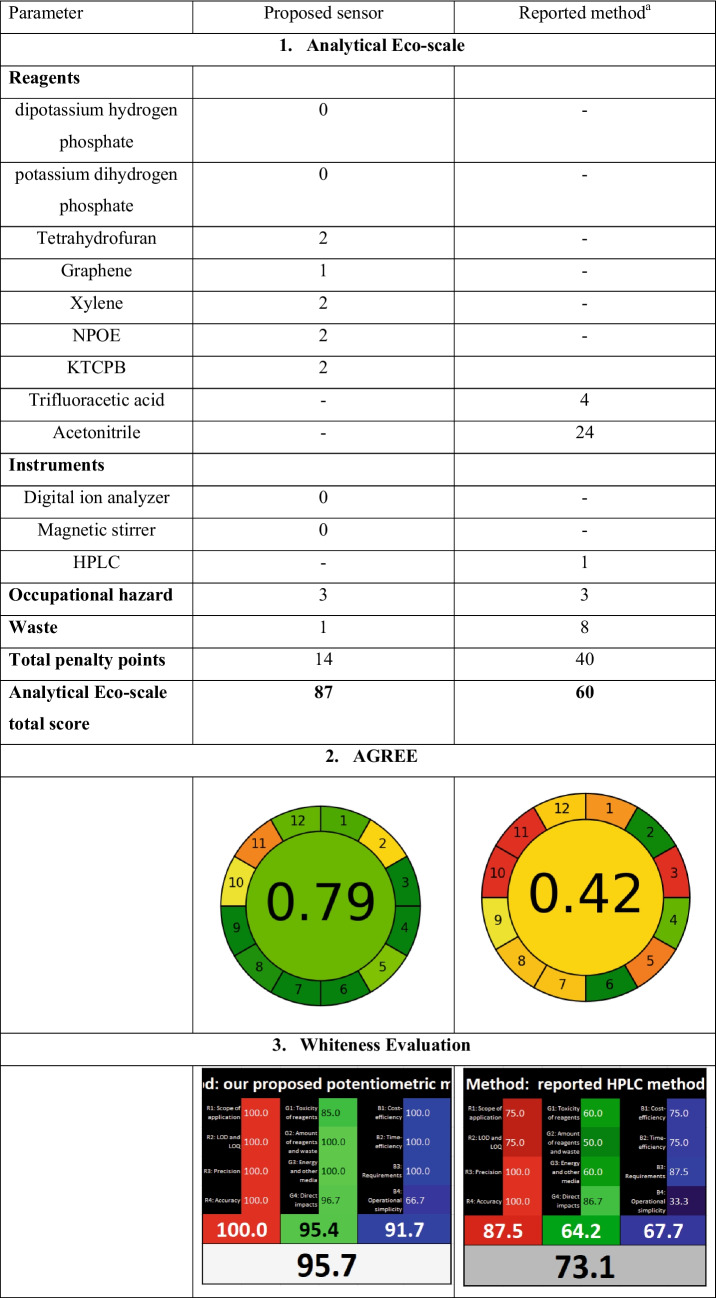
Greenness and whiteness assessment of the proposed method and a reported HPLC method

^a^RP-HPLC method using C18 column (250 × 4.6 mm, 5 µ) with mobile phase composed of 0.1% trifluoroacetic acid and acetonitrile (70:30 v/v) at a flow rate of 1.0 mL/min and UV detection wavelength at 210.0 nm

#### Analytical Eco-Scale

Analytical Eco-Scale is a semi-quantitative approach which assigns penalty points to various steps of the analytical process, which are then subtracted from a base score of 100 to determine the overall greenness of the technique [46]. It classifies methods based on their total score to inadequate (< 50), acceptable (> 50), excellent green (> 75), and ideal green (100). Table 5 presents the detailed penalty points for both the proposed potentiometric methods and a previously reported HPLC method [4]. The findings clearly indicate that the potentiometric approach is far more environmentally friendly compared to the HPLC method, which relies on hazardous organic solvents and produces significant amounts of waste.

#### Analytical GREEnness metric approach (AGREE)

The AGREE is a tool designed to assess the eco-friendliness of analytical methods based on all twelve principles of GAC. The tool produces a visual representation in the form of a clock-like diagram with a color gradient, where the final score is displayed at the center. The score ranges from 0.0 to 1.0, with higher scores indicating a greener method. As the score approaches 1.0, the color of the circle shifts to a darker green tone [47]. For the proposed potentiometric method, the calculated AGREE score is 0.79, which reflects a highly green method when compared with the HPLC-reported method [4] (Table 5). Thus, the AGREE assessment further confirms that the proposed method is green enough for practical application.

#### Whiteness evaluation with RGB12 model

The RGB 12 model consists of twelve algorithms, divided into red, green, and blue categories, each focusing on different aspects of an analytical method [48]. The red category focuses on analytical validation, evaluating the method’s performance. The green category addresses ecological concerns based on the principles of GAC. Finally, the blue category assesses the practical productivity and efficiency of the method. To evaluate the proposed method, the RGB 12 algorithm was used. It involves filling out three tables in an Excel template, which allows for the comparison of various methods. The scores for each principle range from 0 to 100, with 0 representing the least favorable result and 100 the best possible outcome. Once the scores are entered into the template, the results are automatically calculated and displayed in a comparison table. The comparison results, shown in Table 5, reveal that our proposed potentiometric method demonstrates better performance when compared to one of the reported methods [4].

#### United Nations Sustainable Development Goals (UN–SDGs)

The United Nations Sustainable Development Goals (UN–SDGs) provide a global blueprint for achieving sustainability across environmental, social, and industrial domains [49, 50]. In this context, the developed ion-selective electrode (ISE) for IPBr determination aligns with several UN–SDGs, demonstrating how analytical method development can promote sustainability. Its capability to monitor pharmaceuticals quality control aligns with Goal 3 (Good Health and Well-being), while potential application in detecting pharmaceutical residues in very low concentration in water supports Goal 6 (Clean Water and Sanitation). The sensor’s low energy requirement, economic design, and efficient operation contribute to Goal 7 (Affordable and Clean Energy). Decreasing chemical waste and minimizing solvent usage promote Goal 11 (Sustainable Cities and Communities) and Goal 12 (Responsible Consumption and Production). The method supports Goal 13 (Climate Action) by reducing carbon footprint, minimizing energy consumption, and harmful emissions during laboratory operations. It also links to Goal 14 (Life Below Water) through monitoring of pharmaceutical residues and reducing chemical waste, thus contributing to the protection of aquatic ecosystems. Furthermore, method simplicity, sustainability, and ease of transfer between laboratories support scientific collaboration and knowledge exchange, aligning with Goal 17 (Partnerships for the Goals). Overall, the proposed ISE demonstrates how green electroanalytical techniques can achieve the UN–SDGs by combining accuracy, sustainability, and social responsibility.

### Comparison of the proposed sensor with previously reported sensors

The proposed graphene-modified glassy carbon sensor demonstrates excellent analytical performance compared to previously reported sensors, as seen in Table 6. The incorporation of cucurbituril as a host molecule forms a highly stable host–guest complex with IPBr, enhancing both sensitivity and selectivity. The sensor offers a wide linear range (1.0 × 10–6—1.0 × 10–2 M) and a low LOD (6.0 × 10–7 M), with a rapid response time of 5 s. Its solid-contact design ensures superior stability (60 days). The sensor combines sensitivity, speed, and durability for IPBr determination.

**Table 6:** Comparison of the performance of our propsed sensor with the reported electrochemical methods

**Feature**	**Our sensor**	**Reported sensors**		
		Sensor 1 [13]	Sensor 2 [14]	Sensor 3 [15]
**Sensor type**	Graphene-modified glassy carbon electrode	Molecularly imprinted polymer electrochemical sensor	Modified screen-printed potentiometric sensor	PVC membrane electrode
**Linearity range**	1.0 × 10^–6^ to 1.0 × 10^–2^	1.0 × 10^–12^ to 1.0 × 10^–11^ M	1 × 10^−6^ to 1 × 10^−2^ M	1 × 10^–5^ to 1 × 10^−2^ M
**LOD**	6.0 × 10^–7^	2.78 × 10^–13^	2.5 × 10^−7^ M	5.1 × 10^−6^
**Response time**	5 s	10 min	3 s	< 10 s
**Stability**	60 days	5 days	24 weeks	> 2 months

## Conclusion

Two potentiometric sensors were successfully developed for the quantification of the quaternary ammonium compounds Ipratropium Bromide (IPBr) in pharmaceutical dosage forms and spiked human plasma in the presence of its impurity C without any previous separation steps. By incorporating cucurbituril as a receptor-type ionophore, these sensors demonstrated superior selectivity and sensitivity. The use of a liquid-contact platform provided a baseline for sensor performance, while the transition to a solid-contact configuration enhanced portability, durability, and suitability for modern analytical applications, bridging the gap between innovation and practical utility in analytical methodologies. Their ability to accurately determine IPBr in Atrovent® inhalers and plasma samples highlights their potential for therapeutic drug monitoring, particularly in cases of accidental ingestion or overdose. The sensors align with the principles of Green Analytical Chemistry (GAC) and White Analytical Chemistry (WAC), ensuring eco-friendliness and sustainability without compromising analytical performance.

## Supplementary Information

Below is the link to the electronic supplementary material.

Supplementary Material 1 (DOCX 18.2 KB)

## Data Availability

The corresponding author can provide the data upon a reasonable request.
