# Supplementary material for: Eco-friendly cucurbituril-based potentiometric sensors for selective quantification of ipratropium bromide in pharmaceuticals and human plasma
Source: Anal Bioanal Chem. 2025 Dec 22;418(4):1125–37. doi: 10.1007/s00216-025-06265-5 (PMC12901152; doi:10.1007/s00216-025-06265-5)
Supplement: Supplementary file 1 — Supplementary Material 1 (DOCX 18.2 KB) [file 216_2025_6265_MOESM1_ESM.docx]

**Table S1: Raw data (Response) used for Selectivity coefficients calculation of the fabricated sensors towards the interfering ions**

| **Interfering ion (1.0× 10 ^-3^ M)** | **Response** | |
| --- | --- | --- |
|  | **Liquid contact sensor** | **Solid contact sensor** |
| Sodium Chloride | -8 | -14 |
| Potassium Chloride | -9 | -35 |
| Citric acid | -2 | -9 |
| Urea | -13 | -66 |
| Sucrose | -10 | -42 |
| IPBr impurity C | 38 | 29 |

**Table S2: Raw data (Response) used for calibration curves constructions calibration curves constructions**

| **Concentration** | **Response** | |
| --- | --- | --- |
|  | **Liquid contact sensor** | **Solid contact sensor** |
| 1.0 x 10^-7^ | 2 | 21 |
| 1.0 x 10^-6^ | 15 | 50 |
| 1.0 x 10^-5^ | 70 | 106 |
| 1.0 x 10^-4^ | 127 | 164 |
| 1.0 x 10^-3^ | 187 | 218 |
| 1.0 x 10^-2^ | 243 | 280 |

**Table S3: Raw data (Response) used for pH study of the fabricated sensors**

|  | **Liquid contact sensor** | | **Solid contact sensor** | |
| --- | --- | --- | --- | --- |
| **pH** | **1.0 x 10^-3^ M** | | **1.0 x 10^-4^ M** | |
|  | **Response** | | | |
| 2 | 210 | 241 | 142 | 209 |
| 3 | 198 | 236 | 136 | 196 |
| 4 | 194 | 219 | 128 | 163 |
| 5 | 187 | 219 | 127 | 163 |
| 6 | 187 | 218 | 127 | 164 |
| 7 | 187 | 218 | 126 | 164 |
| 8 | 186 | 217 | 126 | 165 |
| 9 | 185 | 216 | 125 | 166 |
| 10 | 185 | 215 | 125 | 166 |
| 11 | 174 | 207 | 115 | 160 |
| 12 | 170 | 198 | 109 | 149 |

**Table S4a : Raw data (Response) used for selectivity study of liquid contact sensor**

| **Concentration** | **NaCl** | **KCl** | **Citric Acid** | **Urea** | **Sucrose** | **IPBR Impurity C** |
| --- | --- | --- | --- | --- | --- | --- |
|  | **Response** | | | | | |
| **1.0 x 10^-5^** | -10 | -15 | -5 | -5 | -14 | 16 |
| **1.0 x 10^-4^** | -10 | -13 | -4 | -8 | -12 | 19 |
| **1.0 x 10^-3^** | -8 | -9 | -2 | -13 | -10 | 38 |
| **1.0 x 10^-2^** | -5 | -8 | -2 | -22 | -8 | 58 |

**Table S4b : Raw data (Response) used for selectivity study of solid contact sensor**

| **Concentration** | **NaCl** | **KCl** | **Citric Acid** | **Urea** | **Sucrose** | **IPBR Impurity C** |
| --- | --- | --- | --- | --- | --- | --- |
| **1.0 x 10^-5^** | -42 | -60 | -16 | -74 | -52 | 21 |
| **1.0 x 10^-4^** | -36 | -54 | -13 | -71 | -50 | 22 |
| **1.0 x 10^-3^** | -14 | -35 | -9 | -66 | -42 | 29 |
| **1.0 x 10^-2^** | -8 | -20 | -2 | -61 | -32 | 35 |
